# Comparing the effects of self- care education by lecture and smartphone application on self-efficacy of patients with thalassemia

**DOI:** 10.1186/s12911-023-02097-4

**Published:** 2023-01-30

**Authors:** Fahimeh Kharaman-nia, Habibolah Rezaei, Narges Roustaei, Peyman Etemadfar, Nazafarin Hosseini

**Affiliations:** 1 Student Research Committee, Yasuj University of Medical Sciences, Yasuj, Iran; 2 Cellular and Molecular Research Center, Yasuj University of Medical Sciences, Yasuj, Iran; 3 Department of Biostatistics and Epidemiology, School of Health and Nutrition Sciences, Yasuj University of Medical Sciences, Yasuj, Iran; 4 Department of Pediatrics, Yasuj University of Medical Sciences, Yasuj, Iran; 5 Department of Nursing, School of Nursing, Social Determinants of Health Research Center, Yasuj University of Medical Sciences, Yasuj, 759195436 Iran

**Keywords:** Thalassemia, Self-efficacy, Self-care, Education, Smartphone application, Lecture

## Abstract

**Background:**

Patients with the thalassemia have a basic requirement for self-efficacy regarding their treatment procedure. The present study aimed to compare the effect of self-care education via a smartphone application and lectures on the self-efficacy of patients with thalassemia.

**Methods:**

In the present quasi-experimental study, 99 patients with thalassemia at Shahid Beheshti Hospital in Yasuj, Iran, in 2019 who were eligible to enter the study, were selected. The block randomization was done with the block sizes of 3, 6, and 9, and participants were randomly assigned to 3 groups: smartphone application (A), lecture intervention (B), and control (c). Smartphone application and lecture interventions were performed for intervention groups A and B, respectively, during 8 weeks. Scherer's self-efficacy measure was used to collect the data at baseline and eight weeks after the intervention. Data were analyzed by SPSS-version 25 software using Paired t-test, Wilcoxon and Kruskal–Wallis tests.

**Results:**

Ninety-nine patients with thalassemia with a mean (SD) age of 25 (6) and 58 females (58.59%) participated in the present study. The results indicated a significant difference in self-efficacy among 3 groups after intervention (*P* = 0.001). However, self-care education with smartphone application revealed a significant increase in the mean (SD) of self-efficacy of the patients with thalassemia 68.36(8.45) compared to the lecture method 62.55 (7.3) (*P* = 0.003) and control 62.09 (6.7) (*P* = 0.001). There were no significant differences among the self-efficacy scores of the patients in lecture intervention and control groups.

**Conclusions:**

The results revealed that smartphone application was a suitable educational software to promote self-efficacy in patients with thalassemia. It is recommended to use smartphone application methods to improve the self-efficacy levels of patients with thalassemia.

**Supplementary Information:**

The online version contains supplementary material available at 10.1186/s12911-023-02097-4.

## Introduction

Thalassemia syndrome is the most prevalent genetic illness [1] caused by a deficiency in one or more genes involved in making globin chains [2], impairing the ability of red blood cells to transport oxygen [3]. Thalassemia and its treatment have consequences and complications, such as damage in the heart, lungs, liver, and endocrine organs in terms of anemia and iron overload, growth retardation, shape changes, bone abnormalities, infertility, and negative mental and social consequences. These problems and uncertainty about the future decrease the quality of life of these patients [4]. Increasing their self-efficacy levels improves the patient's quality of life [5]. Self-efficacy during disease is defined as the person's beliefs about his/ her ability to achieve the desired health outcome [6].

Studies have indicated that patients with higher self-efficacy point out more self-care behaviors, leading to better disease control, improved physical function, and higher quality of life [7]. Self-care in thalassemia patients include nutrition, physical activity, using iron remover, chelation therapy, regular blood transfusion and visits to the doctor, and not smoking [8]. Among the effective factors in developing self-care behaviors in the patients with thalassemia, self-efficacy is a significant factor [9]. One way to improve self-efficacy in the patients with a chronic disease is to provide them with self-care education [10]. Pouraboli et al. [11] reported that thalassemia patients thought that to feel self-efficacy, they needed to understand how to manage their condition and take the best possible care of themselves. Patients may be educated in various ways, including individually, in groups, at their homes with community health nurses, and via smartphone devices [12]. Despite the emergence of newer techniques and expanding knowledge, the lecture method is still considered a common teaching method. The major benefit of this strategy is its capacity to offer knowledge to a large number of learners [13]. If the educator has adequate skill in adopting this strategy, he/she may utilize his/her knowledge, experience, tact, and promote learning among learners [14]. Nonetheless, theorists assert that in terms of the dependence of lecture method on a particular time and place, lecture-based education is no longer successful [15]. Other critiques of the lecture technique include passive learning, disregarding individual variations, and neglecting problem-solving, critical thinking, and other cognitive abilities [16]. Smartphone application (app) technology is one of the foundations of information technology which has entered the education field and introduced smartphone application-based education. This communication device has paved the way for learning at home, work, and on a trip, regardless of time and space, and has detached many limitations and inefficiencies of lecturing [12]. Flexible scheduling of training sessions, the ability to display multimedia content, student and teacher engagement at the same time, the attractiveness of the learning environment, and the potential for deeper learning are all benefits of employing electronic learning over conventional techniques [17]. Software for health-related education has a number of benefits, including affordability, accessibility, and adaptability for various lifestyles [18]. Several studies have been conducted on the effect of different educational methods on self-efficacy of various diseases. In a study, Ndosi et al. indicated the positive effect of patient-based education on improving the self-efficacy of patients with rheumatism [19]. Besides, Tarakmeh et al. revealed the positive effect of training telenursing self-care on self-efficacy of adolescents with thalassemia major [20]. In a study by HasanPour-Dehkordi et al., there was no significant difference among the effects of education via the lectures and self-study on increasing parents' awareness of thalassemia [21]. In another research, Sheikh Abumasoudi et al. indicated no significant difference among the effects of face-to-face (lecture) and electronic training methods on depression, anxiety, and stress of patients with breast cancer [22]. Moreover, a number of studies were conducted on the design of smartphone health applications for chronic diseases. For example, in a study by Choi et al., the smartphone health app improved self-efficacy and increased the knowledge of epilepsy in patients with epilepsy [23]. Though, there are few studies on using self-care smartphone app for patients with thalassemia. In a study by Leonard et al.'s, using Smartphone Health "Selfie" Application led 81% of patients with thalassemia to comply with the medication, increase their knowledge of chelation therapy and decrease their serum ferritin [24]. In the study by Ward et al., the thalassemia self-management app improved adherence to iron chelation and disease self-management [3]. However, the two studies mentioned above were without a control group and at the pilot study level [25]. Additionally, the impact of educating patients with thalassemia how to take care of themselves through a smartphone app on their level of self-efficacy has not been studied. The present study was conducted in order to assess the impact of self-care instruction on patients with thalassemia utilizing lecture and smartphone app techniques. Furthermore, the researchers propose to answer this hypothesis: “Smartphone application has a more positive effect on self-efficacy than the lecture intervention group”.

## Material and methods

### Design and sampling

The present quasi-experimental study was carried out as a pretest–posttest design with a control group from August 2019 to February 2020. The study population were the thalassemia patients with active medical records who were referred to Shahid Beheshti Hospital in Yasuj in 2019. At the beginning of the trial, there were 135 thalassemia patients. Considering limited research population, only 99 patients with thalassemia were eligible for the study.

The inclusion criteria were age (16 years old and above), being literate, and having a smartphone application on which the app could be installed (for the intervention group). The exclusion criteria were patients with behavioral and mental disorders and other underlying medical conditions such as diabetes.

A total of 99 eligible individuals participated in the study and completed the informed consent form. They were randomly assigned to groups A (Smartphone App Intervention), B (lecture Intervention), and C (Control) through the block randomization method. Different block sizes were created by the block randomization process. The individuals were divided into groups at random, with block sizes of 3, 6, and 9. In this strategy, the intervention and control groups each had the same number of samples.

### Instruments

The self-care questionnaire created by Gharaati et al. [8] and the general self-efficacy scale by Scherer [26] was used to gather the data. The Smartphone self-care application was created using the self-care questionnaire, which was used to evaluate needs. It consisted of three sections: demographic characteristics and background information of patients, awareness, and self-care behaviors of patients with thalassemia. The questions on awareness were answered with “True”, “False”, or “I don’t know”. “True” was given a score of 1 and “False” or “I don’t know” were given a zero each. The questions on the patients' attitudes were scored based on the 5-point Likert scale. The patients' replies to the questions on their self-care behavior were always, often, sometimes, and never. According to Gharaati et al. the reliability of the questionnaire was 0.85 for awareness and 0.71 for attitude and performance. Moreover, content validity was used to support the questionnaire's validity [8]. Sherer’s General Self-Efficacy scale was used to assess the self-efficacy level. Imam studied the psychometric properties of English version of Sherer et al.’s 17-item General Self-efficacy Scale. On a 5-point Likert scale, the range score varied from 17 to 85 points. 17 to 35 indicates low self-efficacy, 36 to 57 indicates moderate self-efficacy, and 58 to 85 indicates strong self-efficacy. Psychometric results showed the levels of *r* = 0.74–0.90 for test–retest reliability, *r* = 0.76–0.89 for internal consistency. For reliability, the analysis produced a Cronbach’s alphas, ranged from 0.83 to 0.86 (*p* < 0.0001( [26]. In Iran, Asgharnezhad et al. confirmed its validity and reliability with a Cronbach’s alpha of 0.83% [27, 28].

### Developing Smartphone Self-Care Application

ADDIE model was used to design the educational program and application content, based on which app was developed [29]. According to the model, following steps were taken to create the app: Analysis, Design, Development, Implementation, and Evaluation.

### Analysis

To determine the needs of the patients with thalassemia, the self-care questionnaires were distributed among the patients in both groups at this stage. The questionnaires were collected and analyzed (Additional file 1), which the educational needs of the patients with thalassemia in terms of self-care were identified. The researchers furthermore completed a thorough review of the educational guidelines, textbooks, and accessible resources to evaluate the content and breadth of the program and to identify educational requirements. In addition, a panel was held with some specialists, including hematologists and nurses, to deal with the self-care needs addressed in the app program. Thus, the most important self-care problems (educational needs) of the patients with thalassemia were identified as follows: insufficient knowledge about self-care in thalassemia and its importance, the complications of thalassemia and how to deal with them, the inappropriate attitude of patients toward the thalassemia, nutritional behaviors and diet. Moreover, mental, psychological, and social problems related to thalassemia disease, reduction of physical activity, irregular administration of iron removers, intravenous desferal, and blood transfusion, inadequate attention to the symptoms of hemoglobin deficiency, smoking in a number of patients with thalassemia, and irregular visits by different specialists were acknowledged.

### Design

At this stage, the objectives, educational methods, media, and training strategies were determined. Educational objectives were set based on the needs of patients with thalassemia in self-care. Patients with thalassemia can explain the importance and the components of self-care in thalassemia, the complications of thalassemia and how to deal with them, mention proper diet and nutritional behaviors in thalassemia. In addition, the effects of smoking on thalassemia, and the steps to quit smoking, describing the importance of physical activity in patients with thalassemia and its type and severity, explaining the importance of timely use of iron removers, side effects, and when to use them were determined. Moreover, the importance of timely blood transfusion and blood transfusion care in patients with thalassemia, mentioning the importance of regular visits to different specialists, using the smartphone self-care app, and answering the questions raised, improving mental, psychological, and social problems related to thalassemia in the patients, and finally increase patients' awareness about stress management were recognized.

The education method was smartphone app. The media used in this method included images, text message and motivational videos. Training strategies such as question and answer were applied.

### Development

A smartphone self-care app was created based on the learning objectives and the structure determined in the analysis stage by a professional web developer.

### Implementation and evaluation

The appropriateness of the program was determined by evaluating its efficiency and applicability at the implementation stage [29–31]. The app was given to five participants with a higher level of literacy who were familiar and interested in smartphone software. later on, the educational app was finalized by using their feedback and the necessary modifications were made.

The designed app provided the following features to the users:

#### User registration and login

The initial step after downloading the app was to register user information in order to access its features. The software included three user types: patient, doctor, and nurse. Nurses and doctors each had an individual management panel.

#### Education section

The educational content was designed to increase patient awareness, patient attitudes, and self-care behaviors. In the education section, a set of tutorials on self-care in thalassemia was provided to the users, including thalassemia disease, complications of thalassemia and how to deal with them, general self-care and its importance in thalassemia, such as diet and nutritional behaviors, physical activity, stress management, smoking, iron chelation (training on using chelators, side effects, and time of use), blood transfusion, and importance of regular visits of specialties. In addition, some contents were provided for improving psychological and social issues related to thalassemia, such as self-concept and body image, and creating positive thinking and hope.

The educational content was extracted from relevant sources, such as Guidelines for the Management of Transfusion Dependent Thalassemia [32], Wong Pediatric Nursing Textbook [33], Pediatric Hematology and Oncology Handbook [34], Hemoglobin Disorders Nursing Guide, Comprehensive Handbook of Thalassemia, Comprehensive Package of Thalassemia Care, and other authoritative sources. The department's subject matter experts endorsed the accuracy of the information. The researcher, a nurse with at least 10 years of experience familiar with thalassemia patients, provided the whole instructional material under the supervision of a thalassemia ward doctor.

#### View time of visit

In this section of the app, the time and date of the patient's referral to the ward for medical treatment were presented to him/her. The date of the referral was recorded by a nurse in the app management panel.

#### View laboratory test results

The nurse recorded the laboratory test results of the patients through the admin panel, and each patient could see the details of his/her test results in the test results section. The software assessed the data, and if the test parameters dropped or went up, a notification was sent to the physician’s panel, and a warning message was left for the patient. The physician received the alert on his/her own administrative panel and issued the patient the relevant instructions.

### Intervention

The interventions were self-care education of the patients with Thalassemia by smartphone application and lecture. The outcome of the study was Self-Efficacy of Patients.

*The intervention group A (Smartphone App Intervention)*: Patients in intervention group A were taught in a 90-min session how to work with the application. The application was installed on patients' smartphones, and each patient was given a username and password. A web-based version of the content management system was provided for the central computer. The smartphone app gradually provided educational content to the patients in the intervention group A once in two days for 8 weeks. The users were informed of the introduction of new educational content to the app through a notification.

After the provision of each educational material, the users were asked a number of questions in the daily evaluation section of the application and were given scores (giving scores to the users and ranking them could create a sense of competition and motivation to study the educational content). Users who didn't answer the questions or answered them incorrectly would be reminded to read the tutorial again and answer the questions within a few days. Each instructional resource contained a space for patients to ask questions and get answers concerning that topic to maintain continual communication with patients and address their queries.

*Intervention group B (Lecture intervention)*: Received the same training information through weekly lectures for eight weeks (one session per week) from the researcher and personnel in the thalassemia ward of Shahid Beheshti Hospital of Yasuj, Iran. In this method, question and answer strategies and class discussion were used. The media used in this method included PowerPoint presentation, images, and a booklet. The educational content was presented in eight 2-h sessions, the last 30 min of each were dedicated to questions and answers and sharing the self-care experiences of the patients with thalassemia. During the sessions, the patients were actively involved via the question-and-answer strategy. Besides, the content of each session was given to patients in the form of a booklet.

*The control group (group C)*: The control group only received the routine tutorials. It was attempted during the intervention to control and reduce the relationship among the patients in the control and intervention groups as much as possible.

After completing the tutorials, the patients’ self-efficacy level was re-evaluated to determine the effect of self-care education on it. The post-intervention evaluation was performed using Scherer's general self-efficacy scale 8 weeks after the end of the training course in all study groups (week 16) [8].

### Data analysis

SPSS-25 software was used to process the gathered data. The data were analyzed using descriptive statistics and inferential tests with a significance threshold of 0.05. To assess qualitative data, the Chi-square test was used. The normality of data in the groups was initially evaluated using the Shapiro–Wilk test. Regarding the normal distribution of the data, the parametric paired t-test and non-parametric Wilcoxon and Kruskal–Wallis tests were used as well. Moreover, post hoc multiple comparison test with Bonferroni correction to follow the differences between the groups was used.

## Results

Ninety-nine patients with thalassemia participated in the present study and stayed until the end of the study. The participants included 58 females (58.59%) with the mean ± standard deviation (SD) age of 25 ± 6 years and a health insurance coverage of 74.75%. In terms of education levels, 51 people (51.52%) had a high school diploma or academic education and the others had lower education. The results did not indicate any marked difference among the participants of three groups of smartphone app intervention, lecture intervention, and control in terms of age, sex, education, and health insurance status (*P*-value > 0.05) (Table 1).

**Table 1:** Demographic information of participants with thalassemia

Variable	App	Lecture	control	*P*- value
	N = 33	N = 33	N = 33	
Age (y) M ± SD	24.52 ± 6.76	25.82 ± 5.39	24.12 ± 5.85	0.45 ^Ɨ^
*Sex: N%*				
Male	13 (39.40)	13 (39.4)	15 (45.45)	0.48^¥¥^
Female	20 (60.60)	20 (60.6)	18 (54.55)	
*Education: N%*				
Primary	7 (21.21)	8 (24.24)	4 (12.13)	0.81^¥¥^
Middle school	3 (9.10)	2 (6.06)	6 (18.18)	
Secondary school	5 (15.15)	3 (9.10)	10 (30.30)	
Diploma	10 (30.30)	11 (33.33)	6 (18.18)	
University	8 (24.24)	9 (27.27)	7 (21.21)	
*Medical insurance: N%*				
Yes	25 (75.76)	25 (75.76)	20 (60.60)	0.30^¥¥^
No	8 (24.24)	8 (24.24)	13 (39.40)	

M ± SD: Mean ± SD; N (%): Frequency (percent)

^Ɨ^Analysis of variance (ANOVA) for age variables

^¥¥^Chi- square test

The Shapiro–Wilk test was used in order to check the normality of the data. Before the intervention, the self-efficacy score had a normal distribution in the three study groups. After the intervention, self-efficacy had an abnormal distribution in app intervention and control groups. Moreover, the difference before and after the self-efficacy score in the app intervention group had an abnormal distribution. As a result, analysis of variance was used to compare between groups before the research intervention, and Kruskal–Wallis test was used for inter-group comparison after research interventions (Table 2). Moreover, for intra-group comparison, the Wilcoxon signed rank test was used in the app group and the paired t-test was used in the lecture and control groups (Table 3).

**Table 2:** Comparison of self-efficacy scores between study groups

Group	N	Pre-test	Post-test
		M ± SD Median (IQR)	M ± SD Median (IQR)
App	33	62.73 ± 7.32 64(9)	68.36 ± 8.45 70 (7)
*70(7)*			
Lecture	33	60.3 ± 10.1 62(13)	62.55 ± 7.3 63 (10)
*63(10)*			
Control	33	56.7 ± 10 61(17)	62.09 ± 6.7 64 (6)
*64(6)*			
Test statistics		0.99	15.40
*P*-value		0.37^¥^	0.001^¥¥^

M ± SD: Mean ± SD, was used for Analysis of variance test

Median (IQR): Median (Interquartile Range) was used for Kruskal–Wallis test,

^¥^Analysis of variance

^¥¥^Kruskal–Wallis test, (Significance level *P* < 0.05)

The mean post-intervention self-efficacy score of the smartphone app group was meaningfully higher than those of the lecture (*P* = 0.003) and control groups (*P* = 0.001), according to paired comparisons with Bonferroni correction used to track the differences between the three groups. Nonetheless, the mean post-intervention self-efficacy scores between lecture group and control group were not significantly different (*P* > 0.05)

**Table 3:** Comparison of self-efficacy scores within study groups

Group	N	Pre-test	Post-test	Test statistics	*P*-value
		M (SD) Median (IQR)	M(SD) Median (IQR)		
App	33	62.73(7.32) 64(9)	68.36(8.45) 70(7)	−3.73	0.001^Ɨ^
lecture	33	60.3(10.1) 62(13)	62.55(7.3) 63(10)	−1.80	0.081^ƗƗ^
Control	33	56.7(10) 61(17)	62.09(6.7) 64(6)	−1.89	0.068^ƗƗ^

Mean ± SD: Mean ± SD, was used for paired *t*-test

Median (IQR): Median (Interquartile range) was used for Paired *t*-test

^ƗƗ^Paired t-test

^Ɨ^Wilcoxon signed rank test

Significance level *P* < 0.05

The results indicated no statistically significant difference among the self-efficacy scores of the smartphone app, lecture, and control groups (*P* > 0.05) before the education. On the other hand, the post-intervention scores of three groups were significantly different (*P* < 0.001) (Table 2).

In addition, the results indicated that the self-efficacy mean score of the smartphone app group considerably increased in the post-test compared to the pre-test (*P* < 0.05). Although the mean self-efficacy scores of the lecture group, and control group increased in the post-test compared to pre-test, the differences were not statistically significant (*P* > 0.05) (Table 3).

## Discussion

The present study aimed to compare the effects of self-care education through a smartphone app and lectures on the self-efficacy levels of patients with thalassemia. The present research was a novel study, since no smartphone app was designed for distance education of patients with thalassemia in previous studies, and the researchers found no similar study.

The current study's findings disclosed that the smartphone app intervention group's self-efficacy increased greatly. Mobile health is embraced in this context as a potential player in managing non-communicable and chronic illnesses, even in low- and middle-income countries. This technology has a great potential for empowering patients, health workers, and health system managers. The tools can improve the performance of health workers by providing online guides, reminders, and referral services, and help care for patients via reminders and self-management. [35]: however, the application was equipped with reminders and guidance. Marzuki et al. developed some applications for training colorectal cancer prevention and indicated that accessing information via smartphone was easier and more convenient than attending public places [36]. Similarly, the participants of the present study stated that the advantage of the app was the unnecessity of attending public places to be instructed and the lack of travel expenses and costs. Liu et al. [37] concluded that smartphone application-based self-care interventions were effective tools for managing blood sugar and blood pressure. One of the requirements for empowering an individual’s self-efficacy behaviors is developing self-care habits. Self-efficacy is one of the most essential aspects in the development of self-care habits. An increase in self-efficacy leads to improving previous behaviors, such as adherence to treatment, stress management, physical activity, and its decline leads to an increase in depression and a decrease in self-care activities [9].

In the study by JafarBeglu et al. in Iran, self-care training improved the self-efficacy of patients with diabetes. [38]. Moreover, in the study by Biglar Chopoghlo et al. [39] in Iran, self-care training with telegram improved the self-efficacy of young people with type 1 diabetes. Although above studies have measured the effect of self-care training on the self-efficacy of patients with diabetes, both virtual and face-to-face self-care training approaches have improved self-efficacy in patients. Therefore, self-care training can improve the self-efficacy of patients with thalassemia.

In the study of Fookolaee [17], individuals with thalassemia had better exercise, diet, and stress management as a result of electronic instruction as opposed to typical routine teaching. Furthermore, a study by Munteanu et al. revealed that smartphone app was an effective way to improve asthma control and self-management [40]. Similar to the present study, in the study by Crosby et al. [41], using a self-management smartphone application in the patients with sickle cell anemia increased the involvement of the patients in the self-management strategies of disease. Although self-management was not measured in the present study, it was indicated that improved self-care could lead to self-efficacy and consequently self-management. Likewise, Kazemi Majd et al. [42] indicated that educational text messages increased self-efficacy and adherence to medication in the patients with epilepsy. Similar to the findings of the current research, Tarakmeh et al. [20] study found that after receiving self-care telephone instruction, the self-efficacy of teenage patients with thalassemia major considerably increased. Nonetheless, in their study, the instruction was not based on an app format, rather telephone calls were used to transfer the information. Additionally, a self-care program based on a smartphone application in the patients with Covid-19 enhanced their illness management and health condition in Heidari et al. research [43]. Similar to the current research, Kang et al. [44] found that nursing students' use of Chronic Illness Care Smartphone Apps boosted their understanding of diabetes and hypertension and their sense of self-efficacy. Furthermore, the application improved the assessment of patients and their nursing care by students. However, in a meta-analysis review study by Xu et al., it was stated that while electronic-health based self-management had a significant effect on the fatigue and self-efficacy of cancer patients, it did not improve the quality of life significantly [45]. Similar to the result of present study, in the studies by Mirpuri et al. [46], and Choi et al. [23], the smartphone application increased the meanself-efficacy score of the patients with epilepsy. The smartphone application increased the participants' self-efficacy among patients with various conditions in all of the aforementioned investigations. There are not many research on the creation of smartphone apps for patients suffering from thalassemia. In Leonard et al.'s study, 81% of patients with thalassemia adhered to the medication by using Mobile Health "Selfie" Application. In addition, patients’ knowledge of chelation therapy increased and their serum ferritin had decreased [24]. In the above study, only 11 patients with sickle cell anemia with a lower average age participated. The present study was a before-and-after pilot study without a control group, while in the present controlled trial study, a self-care application was used, and self-efficacy was measured. Furthermore, In Ward's study, the thalassemia self-management app increased iron chelation adherence and disease self-management. However, both of the studies mentioned above were pilot studies and did not measure self-efficacy of the patients with thalassemia. Moreover, a self-care application was used in the present study [3].

The patients in the smartphone app intervention group gained a greater improvement in self-efficacy than those in the lecture and control groups, according to the study's findings. Despite a minor rise in self-efficacy, lecture instruction had no discernible impact. Absoavaran et al. in a study disclosed that the smartphone intervention group using Bluetooth saw a substantial improvement in performance and attitude about breast self-examination in women compared to the lecture group. [47]. Although the nature of disease and outcomes in the aforementioned study were different in two studies, smartphone messages had a greater effect than traditional lectures. However, the findings of the research by Gangi et al. suggested that self-care instruction via lectures might enhance the quality of life for patients with hypertension [48]. Moreover, lectures were predicted to boost self-efficacy, but this did not happen as expected, possibly due to the fact that patients had previously received face-to-face training during their sickness years and the lectures did not enhance patient self-efficacy. This is possibly why lectures were unsuccessful. According to the results of the present study, educational intervention by smartphone application improved the self-efficacy of patients with thalassemia over lectures. In other words, after the research intervention, a marked difference was observed in the self-efficacy score between the smartphone application intervention, and the lecture intervention groups. Therefore, the research hypothesis “Smartphone application has a more positive effect on self-efficacy than the lecture intervention group." was confirmed. In other words, the self-care smartphone app in the patients with thalassemia increased the self-efficacy of patients with epilepsy more than the lecture method.

Similar to the present research, Windisch et al. studied how the Covid-19 knowledge distribution approach for all medical staff at a big university hospital included the usage of the mobile health system. They suggested that mobile health may be a strategy to expand target audience access to trustworthy material, as well as a technique to disseminate time-sensitive medical knowledge. [49]. Therefore, based on the results of this research, it is recommended to use a smartphone to teach self-care and increase the self-efficacy of patients with thalassemia. Using the smartphone application in health systems can not only be help educate the patients, but can be an effective, efficient, and fast method to increase the access of patients with thalassemia and health professionals to valid information, especially during the corona epidemic. Moreover, designing the same smartphone application can be applied to the education, monitoring, and control of the thalassemia and other chronic diseases.

One limitation of the present study was the narrow research population, which might affect the generalizability of the study results. An additional limitation was the possibility of sharing the app among the patients due to their relationship in the hospital and even the long-time friendship they already had. Moreover, the time of educational intervention and follow-up in the present study was short.

## Conclusion

In line with the purpose of present study, the findings indicated that self-efficacy of the patients with thalassemia was not significantly improved by lectures, but rather improved by self-care instruction provided via smartphone apps, and consequently, the research hypothesis was confirmed. It can be said that with self-care training using the smartphone application method, the components of self-care, including the awareness, attitude and performance of patients with thalassemia were improved, and as a result, the self-efficacy of the patients has increased. With further development, and thorough validation of effectiveness of the self-care smartphone application, the use of it in patients with thalassemia can be recommended.

It is suggested that the instruction and follow-up time be increased in future studies. It is as well suggested to study a larger sample size. Moreover, the outcome of the present study was only self-efficacy, and therefore it is recommended to measure the knowledge and self-care of patients with thalassemia, as well as the applicability and feasibility of the app in future studies.

## Supplementary Information

**Additional file 1**. Need assessment based on the self-care questionnaire in thalassemia.

## Data Availability

The datasets generated during and/or analyzed during the current study are available from the corresponding author on reasonable request.

## Abbreviation

app: Application
