# Supplementary material for: Comparing the effects of self- care education by lecture and smartphone application on self-efficacy of patients with thalassemia
Source: BMC Med Inform Decis Mak. 2023 Jan 30;23:21. doi: 10.1186/s12911-023-02097-4 (PMC9885911; doi:10.1186/s12911-023-02097-4)
Supplement: Supplementary file 1 — Additional file 1. Need assessment based on the self-care questionnaire in thalassemia. [file 12911_2023_2097_MOESM1_ESM.docx]

**Need assessment based on the self-care questionnaire in thalassemia.**

**Table S1: Awareness level of self-care in thalassemia patients in Yasuj, Iran.**

| Row | case | Yes  (%) | No  (%) | I do not know (%) | Total  (%) |
| --- | --- | --- | --- | --- | --- |
| 1 | Thalassemia is inherited | 53 | 27 | 20 | 100 |
| 2 | Desferal injection causes excess iron to be removed from the body. | 94 | 1 | 5 | 100 |
| 3 | The most important complication of repeated blood transfusions is the increase of iron reserves in the body. | 86 | 1 | 13 | 100 |
| 4 | Drinking tea or coffee after a meal causes excess iron to be removed from the body. | 86 | 1 | 13 | 100 |
| 5 | Consuming foods such as red meat and liver increases the body's iron reserves. | 94 | 1 | 5 | 100 |
| 6 | Enlargement of the spleen occurs due to the deposition of excess iron. | 35 | 17 | 48 | 100 |
| 7 | Persistent diarrhea and abdominal pain are side effects of Deferal injection. | 6 | 43 | 51 | 100 |
| 8 | Consuming milk and dairy products such as yogurt and cheese increases iron in the body. | 14 | 68 | 18 | 100 |
| 9 | Diabetes (blood sugar) is one of the complications of thalassemia. | 67 | 11 | 22 | 100 |
| 10 | Lack of regular blood and desferal injection causes changes in broad bones. | 67 | 4 | 29 | 100 |
| 11 | The only way to prevent thalassemia is to prevent the birth of an affected fetus. | 63 | 16 | 21 | 100 |
| 12 | Weakness in heart function is one of the side effects of increasing iron in the body. | 78 | 1 | 21 | 100 |
| 13 | A person with thalassemia minor does not need a blood transfusion. | 80 | 6 | 14 | 100 |
| 14 | Atrophy of the gums and jaw bone is one of the complications of thalassemia. | 74 | 7 | 19 | 100 |
| 15 | Hepatitis is one of the complications of blood transfusion. | 52 | 6 | 42 | 100 |
| 16 | By injecting hepatitis vaccine, it is possible to prevent the transmission of this disease through blood. | 49 | 5 | 46 | 100 |
| 17 | Thyroid and parathyroid hypofunction occurs after increasing the amount of iron storage in the body. | 26 | 11 | 63 | 100 |
| 18 | Bone marrow transplant is one of the ways to definitively treat thalassemia. | 79 | 9 | 12 | 100 |
| 19 | In order for the bone marrow transplant to be successful, it must be done before the symptoms of iron deposition appear in the body | 70 | 3 | 27 | 100 |
| 20 | Spleen removal in thalassemia patients must be confirmed by a doctor. | 91 | 3 | 6 | 100 |

%: Percent

The results of the study showed that the mean ± standard deviation of the awareness level in the patients with thalassemia was 12.64 ±3.64 (out of 20 points). Table S1

**Table S2: Nutritional behavior in thalassemia patients Yasuj, Iran.**

| Row | case | Yes, always (%) | Yes, most of the times (%) | Yes, sometimes  (%) | N0, never  (%) | Total  (%) |
| --- | --- | --- | --- | --- | --- | --- |
| 1 | Do you drink tea or coffee immediately after lunch? | 24.7 | 19.8 | 40.7 | 14.8 | 100 |
| 2 | Do you drink tea or coffee immediately after dinner? | 22.2 | 19.8 | 42 | 16 | 100 |
| 3 | Do you eat foods that contain vitamin C such as oranges, lemons, kiwis during Desferal injection? | 6.2 | 13.6 | 51.9 | 28.4 | 100 |
| 4 | Do you check the label of purchased food for iron content? | 7.4 | 8.6 | 21 | 63 | 100 |

(%): Percent

The results of the study showed that 24.7% and 22.2% of the studied patients always drink tea or coffee immediately after having lunch and dinner, respectively. Table S2

**Table S3: Food consumption in thalassemia patients, Yasuj, Iran**

|  | Nutrition | Daily (%) | Weekly (%) | Seldom (%) | Never (%) | Total (%) |
| --- | --- | --- | --- | --- | --- | --- |
| 1 | Tea | 84 | 8.6 | 6.2 | 1.2 | 100 |
| 2 | Coffee | 12.3 | 21 | 30.9 | 35.8 | 100 |
| 3 | Soy | 0 | 33.3 | 53.1 | 13.6 | 100 |
| 4 | Milk and dairy products | 40.7 | 46.9 | 12.3 | 0 | 100 |
| 5 | olive oil | 1.2 | 13.6 | 42 | 43.2 | 100 |
| 6 | Whole grain breads like Sangak bread | 9.9 | 56.8 | 29.6 | 3.7 | 100 |
| 7 | Fish | 4.9 | 59.3 | 29.6 | 6.2 | 100 |
| 8 | Egg white | 12.3 | 56.8 | 21 | 9.9 | 100 |
| 9 | Liver | 2.5 | 19.8 | 53.1 | 24.7 | 100 |
| 10 | Chicken | 14.8 | 64.2 | 19.8 | 1.2 | 100 |
| 11 | Onion | 45.7 | 25.9 | 19.8 | 8.6 | 100 |
| 12 | Date | 6.2 | 37 | 44.4 | 12.3 | 100 |
| 13 | spinach | 0 | 11.1 | 48.1 | 40.7 | 100 |
| 14 | Parsley | 3.7 | 22.2 | 49.4 | 24.7 | 100 |
| 15 | Red meet | 1.2 | 44.4 | 44.4 | 9.9 | 100 |
| 16 | Yolk | 13.6 | 53.1 | 23.5 | 9.9 | 100 |
| 17 | tomato | 45.7 | 44.4 | 8.7 | 1.2 | 100 |
| 18 | Green pepper | 11.1 | 45.7 | 37 | 6.2 | 100 |
| 19 | Dark Chocolate | 11.1 | 12.3 | 38.3 | 38.3 | 100 |

%: Percent

The results showed that 40.7% of patients consume milk and dairy products daily. But 42% of them rarely and 43.2% of them never use olive oil in their diet. The amount of consumption of other food items can be seen in Table S3.

**Table S4: Physical activity in thalassemia patients, Yasuj, Iran**.

| Row | Case | Yes, always  (%) | Yes, most of the times (%) | Yes, sometimes  (%) | N0, never  (%) | Total (%) |
| --- | --- | --- | --- | --- | --- | --- |
| 1 | Have you done activities such as running, aerobics, working with weights in the last month? | 18.5 | 14.8 | 28.4 | 38.3 | 100 |
| 2 | In the past month, have you done anything like brisk walking, cycling, swimming, or similar activities that increase your heart rate a little? | 22.2 | 28.4 | 24.7 | 24.7 | 100 |

%: Percent

The results showed that only 18.5% of patients with thalassemia always have regular physical activity and exercise (at least 30 minutes daily for 4 days per week). Table S4

**Table S5: Chelation therapy in thalassemia patients, Yasuj, Iran**

| Row | Case | Yes, always  (%) | Yes, most of the times (%) | Yes, sometimes  (%) | N0, Never  (%) | Total  (%) |
| --- | --- | --- | --- | --- | --- | --- |
| 1 | Do you use iron removal drugs based on your doctor's advice? | 59.3 | 25.9 | 9.9 | 4.9 | 100 |
| 2 | During the past month, has there been a time when you stopped taking iron-depleting drugs for any reason (pain caused by drug injection - fatigue - impatience - forgetfulness)? | 11.1 | 13.6 | 56.8 | 18.5 | 100 |
| 3 | Based on the recommendation of the doctor and the thalassemia department (medical team), do you visit at the announced time for intravenous Desferal injection? | 27.2 | 19.8 | 30.8 | 22.2 | 100 |
| 4 | During the past month, has there been a time when you stopped intravenous deferral injection for any reason) such as: pain caused by drug injection - fatigue - impatience - forgetfulness)? | 11.1 | 14.8 | 49.4 | 24.7 | 100 |

%: Percent

According to the results, 59.3% of patients with thalassemia always use iron removal drugs based on the doctor's recommendation. However, only 27.2% of the patients, based on the doctor's recommendation and the thalassemia department, have always referred for IV desferal injection at the announced time. Table S5

**Table S6: Blood transfusion in thalassemia patients, Yasuj, Iran.**

| Row | Case | Yes, always  (%) | Yes, most of the times (%) | Yes, Some times  (%) | N0, Never  (%) | Total  (%) |
| --- | --- | --- | --- | --- | --- | --- |
| 1 | Do you go to the thalassemia unit to receive blood based on the blood transfusion program recommended by your doctor? | 93.8 | 6.2 | 0 | 0 | 100 |
| 2 | Are you aware of the symptoms of hemoglobin deficiency that indicate your need for blood transfusion (fatigue, weakness, dizziness)? | 53.1 | 23.5 | 18.5 | 4.9 | 100 |
| 3 | During the past six months, has there been a time when you stopped blood transfusion for any reason (pain from blood transfusion - fatigue - boredom - forgetfulness)? | 4.9 | 0 | 12.3 | 82.7 | 100 |

%: Percent

The results of the study showed that 93.8% of patients with thalassemia follow the blood transfusion schedule. Table S6

**Table S7: The status of the visit of thalassemia patients by medical** **specialists**, **Yasuj, Iran.**

| Row | Medical Specialist | Yes (%) | No (%) | Total (%) |
| --- | --- | --- | --- | --- |
| 1 | Hematologist | 82.7 | 17.3 | 100 |
| 2 | Internal medicine specialist | 40.7 | 59.3 | 100 |
| 3 | Gastrointestinal specialist | 19.8 | 80.2 | 100 |
| 4 | Cardiologist | 69.1 | 30.9 | 100 |
| 5 | Endocrinologist | 23.5 | 76.5 | 100 |
| 6 | Surgeon | 12.3 | 87.7 | 100 |
| 7 | Dentist | 50.6 | 49.4 | 100 |
| 8 | Psychologist | 4.9 | 95.1 | 100 |
| 9 | Optometrist | 32.1 | 67.9 | 100 |
| 10 | Audiologist | 17.3 | 82.7 | 100 |

%: Percent

The results showed that in the past year, the highest number of visits to a hematologist was 82.7% and the lowest was to a psychiatrist (4.9%). Table S7

**Table S8: Smoking status of patients with thalassemia, Yasuj, Iran.**

|  | Case | Yes, always | Yes, most of the times | Yes, sometimes | N0, never |
| --- | --- | --- | --- | --- | --- |
| 1 | do you smoke? | 1.2 | 1.2 | 6.2 | 91.4 |
| 2 | Do you use tobacco? | 2.5 | 1.2 | 12.3 | 84 |

%: Percent

The results of the study showed that only 2.5% of patients with thalassemia always smoke hookah and 1.2% cigarettes.
